# Wealth inequality and weak primary care in the city of São Paulo: ingredients for a dysfunctional and ineffective healthcare system

**DOI:** 10.1590/1516-3180.2017.0104210417

**Published:** 2017-07-31

**Authors:** Rodrigo Diaz Olmos

**Affiliations:** I Professor, Department of Internal Medicine, Faculdade de Medicina da Universidade de São Paulo (FMUSP), São Paulo (SP), Brazil.

I read the Editorial^1^ with interest and agree that preventive interventions should be directed towards social determinants instead of spending huge amounts of healthcare resources on biomedical interventions that may produce more harm than benefit. As the editor pointed out, actions to improve health are mainly outside of the Health Department. Therefore, I too urge the new mayor, Mr. Doria, to adopt science-driven actions to improve health in São Paulo.

I would like to include some ideas that add to the proposal made by the Journal’s Editor. Apart from the factors mentioned in the editorial, there are some other social determinants that significantly affect health outcomes, such as household income, education, wealth inequality and leisure, working and environmental conditions. These, in turn, affect downstream factors, particularly lifestyle choices.^2^ All of these play a more important role in improving health than does medical care. Addressing social determinants of health is an important step towards reaching a healthier society. Social injustice makes people ill and, thus, reduction of these inequities is an ethical imperative.

Medical care is also a determinant of health. However, its importance depends on how it is organized. Medical care based on specialty care, without the coordinating and gatekeeper roles of primary care, tends to be costly, inequitable, medicalizing and worse overall. On the other hand, healthcare based on accessible, continuous, person-centered and integrated primary care tends to produce better health outcomes at less cost.

Adequate investments in the primary healthcare workforce are mandatory, and this means maximizing workforce capacity and stimulating teaching in primary care units. Including teaching within community services as a priority would be cost-effective, since primary care residents trained in this setting would immediately increase the capacity of these services and ultimately expand the primary care workforce.^3^ Investments in high-tech imaging facilities, particularly in the private healthcare sector, on the other hand, are not cost-effective and may produce more harm than good.

Another idea for improving community healthcare services is to change the way in which access is provided within primary care units. Traditionally, scheduling has been based on a variety of appointment types (diabetic care, prenatal care, women’s health, etc.), with a backlog of months and chaotic procedures for triaging patients into crowded office schedules. It produces awkward accessibility to primary care and leads to low patient satisfaction, high emergency department use and overall ineffective primary care. I propose the Advanced Access model, which has increasingly been shown to reduce waiting times within primary care. However, although its principles are potent, they are counter to deeply held beliefs and established practices.^4^ Thus, adopting this model requires leadership and political support.

The last suggestion is to redistribute daily general practice activities from physicians to other healthcare professionals, particularly nurses and pharmacists. This offers a potential solution for primary care supply and demand, thereby empowering other healthcare providers and increasing their satisfaction and effectiveness. Although Brazil seems to stick to old-fashioned, early-20th century practices, there is good recent international evidence for task-shifting of activities from physicians to nurses.^5^

Please, Mr. Doria, read this carefully.
